# The association of mavenism and pleasure with food involvement in older adults

**DOI:** 10.1186/1479-5868-11-60

**Published:** 2014-05-05

**Authors:** Julia Somers, Anthony Worsley, Sarah A McNaughton

**Affiliations:** 1 Deakin University, Centre for Physical Activity and Nutrition Research, School of Exercise & Nutrition Sciences, Faculty of Health, Melbourne Burwood Campus, 221 Burwood Highway, Burwood VIC 3125, Australia

**Keywords:** Older adult, Food involvement, Maven, Pleasure, Behaviour

## Abstract

**Background:**

Food involvement is concerned with the involvement people have in the preparation and consumption of food. Little is known about older people’s food involvement or about the factors which may influence it. Therefore the main aim of this study was to examine food involvement and its associations among older Australians.

**Methods:**

An Internet-based nationwide survey of 1,041 people aged 55 years and over (M = 66 years, SD 6.99) was conducted in 2012. Quota sampling was used to ensure that the age, gender and state of residence of the respondents were representative of the Australian population aged over 55 years. Bell and Marshall’s Food Involvement Scale was administered, along with questions pertaining to socio-demographic, social and hedonic factors.

**Results:**

Overall predictor variables explained 45% (p = <0.0001) of variance in food involvement. Food mavenism and pleasure motivation for food were the factors most strongly associated with food involvement (β = .36; 95% CI .46, .61; p = < 0.0001 and β = .31; 95% CI .78, 1.08; p = < 0.0001, respectively). The predictive ability of demographic factors was reasonably poor.

**Conclusions:**

Food mavenism and pleasure motivation are stronger predictors of Food Involvement than demographic factors. This suggests communication and health promotion opportunities among older people.

## Introduction

Food plays a valuable role in the prevention and management of age-related metabolic diseases [1]. However, older age is a life stage in which people face increasing barriers to meal preparation and optimal dietary intake. A major challenge for public health is how to encourage a rapidly ageing population to prioritise healthy food in their later years. Currently, little is known of the characteristics of people who are more likely to make food a priority in the later stages of life. This study addresses this gap and focuses on food involvement in people aged over 55 years of age.

The construct of involvement has its genesis in consumer behaviour research where it is conceived as a combination of perceived risk and the positive outcomes or inherent rewards of a product or action [2,3]. It has been found to influence cognitive and behavioural responses to products, through memory, attention, cognitive processing and satisfaction [4]. In order to be motivated to engage in a task or activity, people need to feel involvement or personal relevance with a product or situation [5]. Food involvement (FI) is defined as “the level of importance of food in a person’s life” and its utility is in determining the priority people give food ([6], p. 236). The transformation of food is one of the most frequent, time and energy consuming household tasks [7]. A more food involved individual is likely to derive greater pleasure from the activities associated with food and consume a better quality diet [8-11].

It is well established that food plays a valuable role in promoting quality of life and good nutrition is a means of preserving health during the ageing process [12-14]. However, many older people do not eat an adequate diet and many of the leading causes of death in older people are diet related [15-17]. Although older individuals tend to value “proper” meals and “natural” foods, the marketplace is awash with highly processed and energy-dense foods and a plethora of confusing food-related health messages for individuals to decipher [18-21].

Although food plays a valuable role in protecting and maintaining health in older age, little is known of the socio-demographic factors associated with food involvement in older adults. Food preparation, particularly in older generations is still largely a gendered activity with many men reaching older age with little experience or knowledge of the preparation aspects of food [22-26]. Ohly et al. [11] found British men scored more highly on food involvement than women. However this was in a young population (mean age 32.9 years, SD 6.7) of fathers with young children. Although it might be expected that many older women would be more food involved after a lifetime of food provision, some older women can become “fed up” with food-related activities [27-29]. Others however, find new enjoyment in food when they are no longer responsible for food provision to others and have more time to devote to food-related tasks [30,31].

Several studies have shown that FI tends to increase with age [8,11]. For example, Bell and Marshall [6] who designed and validated the main FI scale used in this area, found that being older (up to 65 years) was associated with higher levels of total FI. Education level may also predict food involvement. Indeed Jarman et al. [9], in a large UK study found that 9% of the effect of low education on diet quality was mediated by food involvement [9]. Similarly, domestic living arrangements, such as living alone or with others, may be positively associated with food involvement, as [10] found among a group of university students.

Several social and psychological factors may also be associated with FI. Generally, involvement in social networks facilitates food behaviour [32,33]. For most individuals meal preparation is one part of their social and cultural activities, rather than a nutritional exercise [33,34]. In older age, food preparation and consumption may have major social value involving positive interpersonal relationships and supportive social networks [35,36]. The food choices people make, the occasions when they eat and the preparation methods they adopt are all socially constrained [34,37-39].

Families and friends influence many food and health beliefs and practices, although their influence in older age is relatively unexplored [40,41]. Indeed, reliance on family and friends as information sources may be related to food involvement. Some highly involved people specialise in being sources of information. These ‘market mavens’ are defined as individuals with a general interest in a topic area (like food), who are influential within their social group and willing to share their general knowledge and experience of the topic (food) [42]. Although opinion leaders also tend to be more involved and share many characteristics with mavens, their interests tend to be product specific [42,43]. In the health domain, health mavens appear to be influential disseminators of health information [44]. Therefore, we hypothesise that food mavenism and food involvement may be positively related.

Food involvement may be closely related to enjoyment of food. Health and pleasure factors are strong predictors of diet quality and have previously been identified as factors associated with higher levels of food involvement [6,10,45-47]. As people age, they are increasingly likely to face impediments to their enjoyment of food [48]. Women who are less involved in food transformation (i.e. lower FI), enjoy food less and are more likely to satisfy the food requirements of others before meeting their own food needs [9]. Older Canadians (73 to 87 years of age) who experience a range of barriers to food consumption such as; reduced energy levels, physical disability, lack of appetite and insufficient cooking skills were found to be better equipped to overcome their difficulties, if they prioritised eating well [49]. This involved the conscious allocation of resources (time, effort and money) to food and food was more likely to be prioritised if individual’s derived pleasure from food-related tasks.

**Figure 1:**
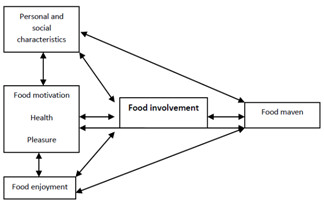
Conceptual model.

The present study explores the direct and indirect characteristics and motivations of greater food involvement in older adults to identify the “food involvement profile” of older adults. As there may be a degree of overlap between the constructs explored, a conceptual model (Figure 1) was developed to better understand the sources of involvement. Three motivational variables were included: health, pleasure and food enjoyment in addition to personal and social characteristics and the mavenism construct. The associations with food involvement are bi-directional to represent a positive feedback loop.

In summary there have been few studies of the predictors of food involvement in older adults, despite this being a life stage in which people are more likely to experience food procurement, eating and meal preparation difficulties ([50,51]; Hughes, Bennett & Hetherington 2004; [52,53]). Identification of the probable influences on food involvement in this age group is likely to facilitate the promotion of healthy and enjoyable food behaviour in later life. Therefore the aim of this paper is to examine the associations between socio-demographic, social and hedonic characteristics and food involvement in a sample of Australians aged over 55 years.

## Methods

The *Food Literacy and You Survey* was an Australian internet-based survey conducted nationally in December 2012. The survey was conducted by Global Market Insights, an international, commercial research panel company. Participants aged over 55 years and residing in Australia were invited by email to join the study. Once the respondents accepted their survey invitations, they were assigned a unique ID to allow access to the survey. Quota sampling was used to ensure the gender, household income and geographic region characteristics of the sample matched those of the general population [54]. Ethical approval was granted by Deakin University, Faculty of Health, Human Ethics Advisory Group, September 2012 (HEAG-H 112_2012).

### Measures

The initial questionnaire was developed from interviews with 16 older adults about their daily food behaviour and from the literature. The questionnaire included questions on food knowledge, information seeking, food enjoyment and motivations, meal preparation, cooking skills, literacy and a measure of diet quality. The questionnaire also included socio-demographic, self-reported health and anthropometric items [55,56]. Where possible, established scales were used and other questions were created for this survey. Cognitive survey pre-testing was conducted using a convenience sample (n = 20). Survey items were modified slightly for clarity, based on participant feedback. One question from Bell and Marshall’s original scale (I do not like to mix or chop food) was modified to read “I do not like to handle (mix or chop) food” due to concerns raised in pre-testing.

#### Outcome variable

##### The food involvement scale

Bell and Marshall’s [6] FI scale was used to capture the perceived level of importance that individual’s place on food. This 12 item instrument includes items on food acquisition, preparation, cooking, eating and disposal (Table 1). The original seven point Likert response scale was used to indicate how strongly people agreed or disagreed with the statements [(Strongly Disagree (1) to Strongly Agree (7)].

**Table 1:** Description of measures including scale items, scale range, scale means and Cronbach alphas

	**Range**	**Mean**	**α**
**Food involvement**^**1**^	**12-84**	**60.05**	**0.73**
**Preparation and eating sub-scale**		**44.15**	**0.757**
**Set and disposal Sub-scale^**		**14.69**	**0.471**
I don’t think much about food each day			
Cooking or barbequing is not much fun			
Talking about what I ate or what I am going to eat is something I like to do			
Compared with other daily decisions, my food choices are not very important			
When I travel, one of the things I anticipate most is the food			
I do most or all of the clean up after eating^			
I enjoy cooking for others and myself			
When I eat out, I don’t think or talk much about how the food tastes			
I do not like to handle (e.g. mix or chop) food			
I do most or all of my food shopping			
I do not wash dishes or clean the table^			
I care whether or not a table is nicely set^			
**Food mavenism**^**1**^	5-35		0.90
I like introducing new foods to my friends and family			
I like helping people by providing them with information about food			
People ask me for information about food			
If someone asked where to get the best information about a particular food or nutrition topic, I could tell him or her where to go			
My friends think of me as a good source of information when it comes to new information about food			
**Pleasure motivation**^**1**^	3-15		0.76
I do not believe that food should always be a source of pleasure,			
The appearance of food makes no difference to me and when I eat			
I concentrate on enjoying the taste of food			
**Health motivation**^**1**^	3-15		0.69
The healthiness of food has little impact on my food choice			
I am very particular about the healthiness of the food I eat			
I eat what I like and do not worry much about the healthiness of food			
**Food enjoyment**^**2**^	6-30		0.71
I used to enjoy the taste of food more than I do now			
A special diet keeps me from eating the food I would like to eat			
Health problems keep me from eating the food I would like to eat			
Money problems keep me from eating the food I would like to eat			
Eating alone most of the time keeps me from enjoying my meal			
Cooking problems keep me from enjoying the foods I would like to eat			
**Social connection**^**3**^	6-36		0.86
**Relative:** Considering the people you are related by either marriage or birth			0.89
How many relatives do you see or hear from at least once a month?			
How many relatives do you feel close to, such that you could call on them for help?			
How many relatives do you feel at ease with that you can talk about private matters?			
**Friendship:** Considering all of your friends including those living in your neighbourhood			0.87
How many friends do you see or hear from at least once a month?			
How many friends do you feel close to, such that you could call on them for help?			
How many friends do you feel at ease with that you can talk about private matters?			

^1^Strongly disagree to strongly agree, ^2^very true to not at all true, ^3^none, one, two, three or four, five to eight or more than nine contacts.

^Set and disposal items.

The items were scored and summed according to Bell and Marshall’s instructions and the resulting scale had a range of 12 to 84. The internal reliability for this data was good (Cronbach’s alpha = 0.73). Factor analysis conducted by Bell and Marshall produced two sub-scales. One titled ‘set and disposal’ with a range of 3–21 and another named ‘preparation and eating’ with a range of 9–63. A principal component analysis (with varimax rotation) was conducted on the 12 item FIS to confirm the (S&D, P&E) factors identified by Bell and Marshall [6].

#### Independent variables

##### Social connectedness

Social connectedness was measured using [57] Social Network Scale (LSNS-6). This six item scale specifically was designed for older populations to quantify family and friendship ties and identify those at risk of social isolation (Cronbach’s alpha = 0.83). The instructions asked respondents about their frequency of contact, feelings of closeness and sense of ease with family and friends. The response scale allowed respondents to record none, one, two, three or four, five to eight or more than nine contacts. The items were summed to form a scale (the present scale’s Cronbach’s alpha = 0.86, Table 1). A score of less than 12 indicates a high risk of social isolation and a score of 12 or more, indicates a low risk of social isolation.

### Food mavenism

The five item food maven scale was adapted from Feick and Price’s [42] market maven scale. The instructions were modified to ask about “food or food information”, rather than “products” in the original. A seven point Likert response scale was used (strongly disagree (1) to strong agree (7)) and the summed score range was 5–35. The scale had high internal reliability (Cronbach’s alpha = 0.90; Table 1).

### Food enjoyment

Food enjoyment was assessed by the food enjoyment scale for older adults, a six item instrument which used a five point response scale (very true (1) to not at all true (5); Table 1) [58]. The scale items are based on sensory enjoyment, the impact of dietary restriction and oral, financial, social and functional limitations [58]. One question was modified to read “health problems keep me from eating the foods I would like to eat” rather than “mouth or teeth problems”. As all items were negatively worded, the scale was reversed. A composite score of 30 indicates the highest level of food enjoyment and a score of 6 the lowest (Cronbach’s alpha = 0.71).

### Health and pleasure motivation

Health and pleasure motivation was measured with six items from Roininen et al’s [59] Health and Taste scales. The general health interest items dealt with interest in eating healthily, whereas the pleasure items are concerned with the importance of deriving pleasure from food. Three items with the highest factor loadings were taken from the pleasure sub-scale and three items from the general health interest sub-scale (Cronbach’s alpha = 0.76). A seven point likert response scale was employed (disagree strongly (1) to agree strongly (7), Table 1).

### Demographic variables

Details of several socio-demographic variables which might influence food involvement were collected. These included; age, sex, education level (left school at age 16, left school at age 18, Bachelor degree/Diploma/Certificate, Postgraduate degree), marital status (married/de-facto, separated, divorced, widowed, never married) and household size (number of people in household). Frequency of meal preparation (My meals are most usually; prepared by me, prepared by me with assistance from others or prepared by others) and time spent in meal preparation yesterday (none, <15 minutes, 15 minutes) were self-reported [7].

### Health variables

Self-reported health (excellent, very good, good, fair, poor) [60] and self-reported height and weight, which have previously been validated for use in older subjects were measured [55].

### Statistical analysis

Descriptive statistics were used to describe the socio-demographic characteristics of the study participants. Based on the work of Bell and Marshall [6], the food involvement scores were split into tertiles (high = 66+, medium = 56–65 and low = < 55) in order to examine associations between FI and socio-demographic characteristics using chi-square test for proportions. The analysis was also conducted with a split data file (men, women) to compare sex differences. Socio-demographic variables were coded; non-tertiary or tertiary educated, married/de-facto or single, one or more person households and self-reported health good or poor. The measure for social connection was coded <12 “high” risk of social isolation and ≥12 “low” risk of social isolation [57].

Demographic variables which were significantly related to the food involvement scale were then input into multiple linear regression analysis to determine independent effect (Model 1). Social, hedonic and health variables were then entered into the multiple regression model (Model 2). All statistical analyses were performed using SPSS (Version 20) [61].

## Results

### Characteristics of the sample

A total of 1041 people took part in this survey and approximately half of participants were men (Table 2). The mean age of participants was 66 years (SD, 6.99) and the age range was 55 to 88 years. Over two thirds of the subjects were married and more men than women were currently in relationships. The sample was highly educated with 51.6% of participants having bachelor degree level education or higher, compared with 21% of the Australian population aged 55–64 years who have attained tertiary education [62]. A large proportion of participants had retired (n = 637) and the mean age for retirement was 60.4 years (SD 7.7). Overall, 49.4% of subjects had a household income of less than $40,000 per annum.

**Table 2:** Associations between tertiles of the food involvement score and socio-demographic characteristics of adults aged >55 years (n = 1041)

			**Low <55 (n = 349)**	**Medium 56–60 (n = 335)**	**High 66+ (n = 357)**		
	**n**	**FI mean**	**%**	**%**	**%**	***X***^***2 ***^**(Pearson)**	***P *** **value**
**Sex**						53.73	<0.0001
Male	519	57.10	43	34	23		
Female	522	62.37	24	36	40		
**Age**						1.79	0.408
55 – 64 years	472	60.39	32	31	36		
65 years and over	569	59.74	35	33	33		
**Education**						9.08	0.01
Non-tertiary	504	59.25	35	38	27		
Tertiary	537	60.79	32	32	36		
**Social connection**^**1**^						17.11	<0.0001
High-risk social isolation	76	55.13	54	30	16		
Low-risk social isolation	965	60.43	32	35	33		
**Marital status**						11.46	0.003
Partnered	708	59.22	36	35	29		
Single	333	61.80	27	35	38		
**Household size**						10.83	0.004
1 Person	246	62.00	25	37	37		
2 or more people	795	59.44	36	34	30		
**Perceived health**						12.27	0.002
Excellent - Good	725	60.93	31	34	35		
Fair - Poor	316	58.02	39	37	24		
**Usual meal preparer**						80.27	<0.001
Yes	805	62.17	27	33	40		
No	236	52.78	57	28	15		
**Time spent in meal preparation**						104.77	<0.001
No meal preparation	232	53.83	57	28	15		
<15 minutes	118	57.45	47	26	27		
>15 minutes	691	62.58	24	34	42		

^1^< 12 high risk of social isolation, ≥12 low risk of social isolation.

The scale items are shown in Table 1, together with the mean scale scores, the range and scale Cronbach alphas for this study population. The scores for food involvement ranged from 23 to 84 and the mean score was 60 (Table 1). A summary of the statistically significant associations between demographic variables and the Food Involvement Scale (FIS) are shown in Table 2. As previously identified, men were less food involved than women, as were single people and those who did not usually prepare the main meal. The time spent in meal preparation was also positively associated with food involvement and individuals at high risk of social isolation were not as food involved as those at less risk. There was a significant association between non-tertiary and tertiary education levels and food involvement *x*^2^ (2) 9.08, p = 0.01. Food involvement increased with educational attainment (Table 2). Tertiary educated respondents had higher FI scores than the other respondents (36% vs 27%, P < 0.01).

Overall, the predictor variables explained 45% of the variance in food involvement (Table 3). The strongest predictors of food involvement were food mavenism and pleasure motivation for food. Enjoyment of food was positively associated with food involvement. The standardised coefficient for gender was .21 (p = < 0.0001) in model 1, after the addition of intrinsic variables (mavenism, pleasure, health and enjoyment), gender remained significantly associated, however the standardised coefficient reduced to 0.09 (p = 0.0001).

**Table 3:** Summary of the hierarchal multiple regression of associations between socio-demographic, social and hedonic variables with food involvement scores

	**Model 1**^**^**^	**SE**	**T**	**P**	**Model 2**^**^**^	**SE**	**T**	**P**	**R**^**2**^
Constant			28.62	<.0001			6.12	<.0001	
Male or female	.21	.61	7.03	<.0001	.09	.49	3.64	<.0001	.052
Marital status (single, not)	.10	.66	3.24	.001	.14	.52	6.03	<.0001	.062
Self-rated health	-.14	.30	-4.78	<.0001	-.01	.25	-.54	.59	.080
Education	.09	.38	2.92	.004	-.00	.30	-.08	.94	.089
Food maven					.36	.04	14.03	<.0001	.301
Pleasure motivation					.31	.08	12.06	<.0001	.419
Health motivation					.17	.06	6.45	<.0001	.438
Food enjoyment					.12	.06	4.95	<.0001	.449

Model 1 R^2^ = .089, F (4, 1036), p = <0.0001, Model 2 R^2^ = .449, F (4, 1032), p = <0.0001.

^^^Standardised coefficient.

The impact of the demographic variables on food involvement was low, with only gender and marital status being significantly associated. Age and education, were not significantly associated with FI in the regression model 2 (Table 3).

## Discussion

To the best of our knowledge this study is the first to explore predictors of food involvement in older adults. Food mavenism and pleasure motivation were much stronger predictors of food involvement than the socio-demographic variables, such as age, gender and education. These findings are consistent with those of Jarman et al. [9] who showed that negative affect (poor mood, low energy and feelings of stress) was inversely related to FI. Although the constructs measured in this study were different, they clearly show the dominant influence of intrapersonal factors on FI. One of the variables assessed in the present study, food enjoyment, was specifically related to problems associated with food in older age. The mavenism construct is new in the context of food studies.

The single most important and novel predictor of food involvement in the present study was food mavenism (Table 3). The association was expected as food involvement relates to the priority and interest an individual ascribes to food, combined with an overall willingness to share knowledge and experience of food. Both FI and mavenism signify engagement with food. Food has both oral and literate cultures of communication, however the sharing of food information through recipe exchange, tips on preparing unfamiliar foods or cooking techniques, diet strategies and information on food products as social exchanges are relatively unexplored in the literature [63,64]. Evidence from the health literature has shown that mavens are important disseminators of health information, although the information they dispense may not necessarily be accurate [44]. Similarly, food mavens may be important purveyors of food information in older age as people experience physical, social and emotional change that impact food behaviour, but the quality of information shared is unknown.

Mavenism shares some characteristics with the opinion leader construct [43]. Opinion leaders guide the uptake of innovation through product specific knowledge, whereas mavens are driven by a desire to help others and the sense of pleasure they derive from the action of general information sharing [43]. A food maven will therefore have a “propensity to communicate” about food [65]. This may involve talking with people about food management, planning, purchasing, preparation and eating or it may include discussions around food production, environmental considerations, health benefits of foods or issues of cost, access and equity [63,66]. Although this does narrow the maven concept from “all” products to “food”, the construct retains its polymorphous or general influence, rather than the product specific approach associated with opinion leadership [67].

Pleasure from food was also associated with food involvement. Pleasure is highly personal and includes; taste, satiety and enjoyment [68,69]. FI was stronger in meal preparers than those who do not routinely prepare meals and in individuals spending more time in meal preparation. This suggests that older individuals who value pleasure from food in terms of taste, appearance and enjoyment are more likely to engage with food and in meal preparation tasks. Food pleasure is not only obtained through eating, but many individuals also derive pleasure from the social aspects of providing food to others [70]. This too is vulnerable to change in older age as children leave home and relationships change. Evidence suggests this can bring greater enjoyment of food to some as they are free of restrictions associated with preparing food for others, whereas others sorely miss the social companionship of food [71]. The present findings indicate the importance of emphasising the pleasurable aspects of food preparation and consumption in older age.

The association of food enjoyment with food involvement might be expected as people are more inclined towards an activity or object that they perceive they will derive some pleasure from [72]. Food apathy is a significant barrier to maintaining dietary adequacy in older age [28,49,73]. Consistent with this, we found that as the ability to enjoy food declined, so did the degree of food involvement. Previous works have identified enjoyment of meal preparation and the food-related pleasures of taste and smell as conducive to greater engagement with food [7]. These intrinsic factors related to the hedonic appreciation of food and food involvement are at risk with physiological, social and emotional changes frequently associated with increasing age [74,75]. It is likely that if the pleasure of eating declines and meal preparation becomes burdensome, then the priority given to food lessens. More research is required to confirm and extend these findings.

This study extends previous findings that suggest health motivation is an important predictor of engagement with food in older age [27,76]. In previous research, health motivation appears to be inversely related to consumption of convenience foods and is positively associated with consumption of fresh foods such as vegetables [77,78]. The present findings show that motivation and food involvement are positively related to each other. This supports the view that both health motivation and food skills (indexed in the FI scale) are required for individuals to consume healthy food [47,79].

The social mechanisms influencing food and health behaviour in older age are complex and include potential influencers such as social connection, social support, relationship quality, commensality and social networks [33,80,81]. Contrary to studies which have found a positive relationship between social connectedness and food behaviour in older age, the social network measure used here was not associated FI. This is unlikely to be due to the particular characteristics of the scale as it assessed both family and friendship ties and the extent of social contacts [82]. However, the FI scale measures the *preparatory aspects* of food (such as handling produce, cooking, setting the table and clean-up activities) which are quite different from the focus on food *consumption*, found in much of the literature [22,25,35]. This may be partially responsible for the observed discrepancy with the literature.

The often discussed gender divide in food related tasks was reflected in the findings of this study. Gender was associated with food involvement and women were more food involved than men. Although gender remained significant in the final multivariate model, the results suggest that previous findings of gender differences in food behaviour may have been due to the higher FI of women. This may also account for greater FI of single compared to partnered respondents. More food involved women than men were single. Similarly, single men were also more food involved than their cohabiting counterparts. Previous studies indicate that older men coming to food preparation later in life, with little prior food experience, tend to struggle with the unfamiliar processes involved [23,24].

In contrast to previous studies, educational background was not predictive of FI in the final multivariate model. The initial influence of educational background was substantially reduced when the social and hedonic variables were entered into the regression model, suggesting that educational background may influence FI through these variables. A similar process was reported by Wardle et al. [83], who found that nutrition knowledge mediated the effects of educational background on fruit and vegetable consumption.

### Limitations and strengths

The cross-sectional design of the current study prevents causal inferences being made on the basis of these findings. Our model and hypothesis were based on the existing literature and a number of potential confounders were considered as part of this model, however residual confounding may be present due to confounders not measured in our study. Potential confounders including negative affect (mood and interest in life) and ethnicity need to be considered in future research [9,46]. The proportion of highly educated participants and use of an online survey population may also limit the applicability of these findings to other populations. Longitudinal or experimental studies, based on random population samples are needed to confirm and extend the main findings from this study. Further research is required to examine whether the predictors of FI identified in this study apply in other older, ethnically diverse populations and whether food involvement influences dietary intake. Exploration of the quality and influence of information disseminated by more food involved individuals is also an area worthy of further enquiry.

The authors concede that the constructs under investigation here (food involvement, food mavenism, pleasure motivation, health motivation and food enjoyment) undoubtedly overlap each other. However, in terms of correlations, no violations of multicolliniarity were detected. The roles of these concepts in specific food behaviour such as food safety and cooking is worthy of deeper examination in the future. Food mavenism is a previously unexplored construct in FI. Further clarification of the food maven scale, to focus on cooking and meal preparation, rather than “food”, may increase its predictive ability. It is not possible in this cross sectional study to rule out reverse causality between food involvement and predictor variables. That is, food enjoyment or mavenism could in fact cause or influence food involvement and further longitudinal research would clarify this.

### Implications for the promotion of healthy eating in older age

The main novel findings of this study are the strong association of mavenism, pleasure and food enjoyment with FI. Therefore, efforts might be made to identify and provide training to food mavens in older populations. This would provide valuable communication channels with different cultural groups and individuals with varying levels of interest in food. These results also indicate the importance of emphasising the pleasurable aspects of food to older adults. This could be achieved through the provision of food-related social opportunities and strategies to more easily enable people to prepare simple, but tasty meals and snacks. Opportunities to build confidence in food skills in those with little previous experience could be a key strategy to engaging less involved individuals with food. This could involve classes identifying foods, appropriate cooking methods and flavour combinations.

### Conclusion and implications for practice

This study showed food mavenism and enjoyment are stronger predictors of food involvement in an older population than socio-demographic variables. The efficacy of nutrition interventions among older people may be increased through greater focus on food enjoyment and on the identification and training of food mavens.

## Competing interests

The authors declare that they have no competing interests.

## Authors’ contributions

JS conducted the analysis and drafted the manuscript. All authors were involved in critically revising the manuscript, and have given their approval for the submitted manuscript.
